# Impacts of an Invasive Non-Native Annual Weed, *Impatiens glandulifera*, on Above- and Below-Ground Invertebrate Communities in the United Kingdom

**DOI:** 10.1371/journal.pone.0067271

**Published:** 2013-06-28

**Authors:** Robert A. Tanner, Sonal Varia, René Eschen, Suzy Wood, Sean T. Murphy, Alan C. Gange

**Affiliations:** 1 CABI, Egham, Surrey, United Kingdom; 2 CABI, Delémont, Switzerland; 3 School of Biological Sciences, Royal Holloway University of London, Egham, Surrey, United Kingdom; Jyväskylä University, Finland

## Abstract

Vegetation community composition and the above- and below-ground invertebrate communities are linked intrinsically, though few studies have assessed the impact of non-native plants on both these parts of the community together. We evaluated the differences in the above- (foliage- and ground-dwelling) and below-ground invertebrate communities in nine uninvaded plots and nine plots invaded by the annual invasive species *Impatiens glandulifera*, in the UK during 2007 and 2008. Over 139,000 invertebrates were identified into distinct taxa and categorised into functional feeding groups. The impact of *I. glandulifera* on the vegetation and invertebrate community composition was evaluated using multivariate statistics including principal response curves (PRC) and redundancy analysis (RDA). In the foliage-dwelling community, all functional feeding groups were less abundant in the invaded plots, and the species richness of Coleoptera and Heteroptera was significantly reduced. In the ground-dwelling community, herbivores, detritivores, and predators were all significantly less abundant in the invaded plots. In contrast, these functional groups in the below-ground community appeared to be largely unaffected, and even positively associated with the presence of *I. glandulifera*. Although the cover of *I. glandulifera* decreased in the invaded plots in the second year of the study, only the below-ground invertebrate community showed a significant response. These results indicate that the above- and below-ground invertebrate communities respond differently to the presence of *I. glandulifera*, and these community shifts can potentially lead to a habitat less biologically diverse than surrounding native communities; which could have negative impacts on higher trophic levels and ecosystem functioning.

## Introduction

Globally, non-native invasive plants are the most important group of invasive species in terms of number of species and the scale of their impacts on natural environments [1]. However, impact studies of non-native plant species at the community level are poorly represented in the current literature [2], [3], [4]. Where studies have been conducted, the majority have focused on the impact of invasive plants on natural vegetation [5], [6], [7], [8], [9].

When non-native plants invade an environment, they are capable of outcompeting native plant species through either direct [10], or indirect competition [11], [12]. Often non-native invasive weeds form monotypic stands which are of little value to native invertebrate species as a direct food source [13] or, consequently, for prey species [14]. The change in vegetation species composition and structure, resulting from a non-native plant invasion, may alter the invertebrate community composition, which can have knock-on effects at higher trophic levels [15], [16]. Gerber et al. [17] studied the impact of *Fallopia* species on plant species richness and invertebrate populations in Europe and showed that the invaded habitats supported fewer plant species, coupled with a lower abundance and species richness of invertebrates compared to uninvaded plots.

The majority of impact studies have been conducted in an above ground context [18]. Where studies have been conducted on below-ground invertebrates, the results are often conflicting depending on study systems and species. For example, Rudd [19] studied the potential impact of *Lonicera* x *bella* Zabel, an invasive honeysuckle species in the USA, on soil invertebrate diversity using pitfall traps and soil cores, and showed there was no effect of the non-native plant species on soil invertebrate diversity or abundance. In contrast, Belnap and Philips [20] showed an increase in below-ground invertebrate species richness in areas invaded by *Bromus tectorum* L. in south-eastern Utah, USA. Below- and ground-dwelling herbivores are more generalist plant feeders compared to foliage herbivores that specialise on one or a number of closely related species [21]. The varying dominance of different functional feeding groups between trophic levels may account for the more pronounced negative response of above-ground invertebrates to the occurrence of a non-native plant species [22], [23].

It is well known that the above- and below-ground communities are linked through complex interactions [24], [25], [26]. Through herbivory, above-ground invertebrates regulate resources entering the below-ground community, which (indirectly) affects below-ground detritivores [27]. Below-ground herbivores influence the structural composition of above-ground vegetation communities [28], [29] by promoting some plant species over others [30], [31]. Soil detritivores break-down organic plant material and thereby cycle nutrients through the ecosystem to the above-ground community [32].

Plant invasions can disrupt the linkages between the above- and below-ground communities. Invasive plants can alter the resources entering the soil which can have a direct impact on the below-ground detritivore community, and an indirect impact on native plant performance, and consequently on the above-ground invertebrate community [25]. Thus, in order to ascertain ecosystem impacts of non-native plant species, it is important to evaluate both their above- and below-ground impacts, especially in the context of habitat management and ecosystem restoration [33], [34].

The focal species in our study was *Impatiens glandulifera* Royle (Balsaminaceae), a non-native highly invasive annual species that has spread rapidly throughout the UK [35], mainland Europe [36] and North America [37], since its introduction from the foothills of the Himalayas at the beginning of the 19^th^ century. *Impatiens glandulifera* is now the tallest annual plant species in Europe, attaining a height of up to 2.5 m [35]. In the UK, it is predominantly a weed of riparian habitats [35], though it will flourish in damp woodlands and waste grounds [38]. *Impatiens glandulifera* has been shown to displace native vegetation when the cover is high, though often those species affected are widespread ruderal species [7]. In turn, a reduced cover of native vegetation may affect invertebrate communities, which are reliant on native plant species.

To-date, there are few studies that have evaluated the impact of *I. glandulifera* on invertebrate communities. Beerling and Dawah [13] compared the British invertebrates associated with *I. glandulifera* to those of *Fallopia japonica* Houtt (Ronse Decr.), another non-native species, and showed that of the two invasives, *I. glandulifera* harboured a higher above-ground invertebrate diversity. However, we are unaware of any studies that have evaluated the impact of an annual invasive non-native species on above- and below-ground invertebrate communities, and related this to natural vegetation. Therefore, any research in this field could aid the prioritisation of weed targets for control. As an annual, *I. glandulifera* has the potential to change seasonal and inter-annual vegetation dynamics, due to variation in cover, which may be different from the impacts seen by perennial non-native species which have the tendency to form permanent monospecific stands [17]. *Impatiens glandulifera* populations are influenced by climatic conditions, density dependent mortality and abiotic disturbances such as flooding [5], [35]. Fluctuations in the cover of *I. glandulifera* may have an impact directly on the cover of native plant communities [7], and hence the invertebrate community [13].

The objective of our study was to determine if *I. glandulifera* has an impact on the associated invertebrate community, by comparing invaded and uninvaded plots. In particular, we set out to evaluate the impact of *I. glandulifera* on communities both above ground and below ground. The specific hypotheses we tested were:

1. The presence of *I. glandulifera* has a negative impact on invertebrate communities by displacing native invertebrates.
2. The above-ground invertebrate community shows a greater negative response to the occurrence of *I. glandulifera* than the below-ground community.
3. The invertebrate communities respond to the seasonal fluctuations in the occurrence of *I. glandulifera*.

## Methods

### Site Selection and Vegetation Sampling

Harmondsworth Moor is a 135 ha public parkland situated in the county of Middlesex, UK (N 51° 29′ 582, E 00° 29′ 023). Two rivers run through the park, the River Colne, running south of the eastern side of the park, and the River Wraysbury, running south of the western side of the park. *Impatiens glandulifera* has been established at Harmondsworth Moor for over a decade (personal communication, Paul Jarvis, Park Warden). After a preliminary survey of the occurrence and absence of *I. glandulifera* in the park in late April 2007, experimental plots were selected systematically within infested and uninfested areas. Eighteen experimental plots were selected each measuring 20 m by 20 m, nine invaded by *I. glandulifera*, where the percentage occurrence of *I. glandulifera* was no less than 60% of the total area, and nine uninvaded plots where the vegetation composition comprised of predominantly native plant species. All experimental plots were at least 30 m apart, but otherwise similar in their position to a river and thus had the same potential to be invaded by *I. glandulifera*. All experimental plots were selected to be away from the main public pathways and therefore any trampling effects and other human related disturbance was kept to a minimum.

Vegetation cover was assessed in July 2007 and 2008 at each experimental plot. Six 1 m^2^ quadrats were placed randomly within each plot. Vegetation composition was recorded as the percentage cover of individual species estimated with the aid of a 10 cm^2^ string grid within the 1 m^2^ quadrat.

### Invertebrate Sampling

The invertebrate communities were sampled monthly over two seasons from May to September inclusive (2007), and May to August inclusive (2008). All invertebrate sampling was conducted during a two-day period in each month and sampling was carried out at least 28 days from the previous months sampling in each year.

The foliage community of invertebrates was sampled using an aerial suction sampler (reverse leaf blower, JCB Co. Ltd., UK) [39]. For each plot, a sample consisted of a one-minute period during which the collector moved throughout the plot directing the aerial suction sampler in vertical and horizontal directions to encompass the structure of the vegetation. This was replicated six times throughout each plot on each sample date.

The ground-dwelling community was sampled using a Vortis suction sampler (Burkard Manufacturing Co. Ltd, Hertfordshire, UK). The Vortis is a quantitative method for sampling invertebrates as the area sampled is the aperture of the sampling tube; this provides an advantage over pitfall trap methods, as the latter do not sample from a defined area [40]. For each plot, the ground-dwelling invertebrate community sample consisted of six ten-second vacuums per sample, where the collector remained in the same location and turned through 360°, 60° at a time, sampling six equally distant areas. This was replicated six times for each plot, with the location of the sampler randomly selected. In total, an area of 0.6984 m^2^ was sampled for each plot on each sampling date.

To sample the below-ground invertebrate community, a bulb planter (10 cm diameter and 25 cm in depth) was used to extract soil from beneath stands of *I. glandulifera* and native vegetation. One soil sample was taken at random from each plot on each sampling date. Below-ground invertebrates were extracted from the soil cores using Berlese Tullgren Funnels (Burkard Manufacturing Co. Ltd., Hertfordshire, UK). Each soil sample was placed in a separate funnel, covered with plastic wrap to prevent invertebrates escaping upwards. A light source was installed above the funnel to instigate the movement of the invertebrates into preservation containers below. All invertebrates were preserved in 70% alcohol prior to identification.

In total, 139,923 invertebrates (foliage-dwelling 8,709; ground-dwelling 128,337; below-ground 2,877) were sorted into taxonomic groups during this study. With the exception of Diplopoda and Chilopoda, which were identified to class, all invertebrates were identified to order or lower divisions. For the foliage invertebrate community, the abundance of Coleoptera, Auchenorrhyncha (Hemiptera), and Heteroptera (Hemiptera) were recorded. These groups were chosen as the majority of canopy species within these groups are phytophagous and may potentially feed on *I. glandulifera* as opposed to just using the species as a resting place. Araneae were selected as their abundance suggests the presence of prey items [41]. Coleoptera and Heteroptera from the foliage community were identified to morphospecies within families to ascertain a measure of species richness. For the ground-dwelling and below-ground communities, all invertebrates were identified to distinct taxa. All taxa were categorised into functional feeding groups, based on the predominant feeding preferences of the species in each group with the exception of larvae in the below-ground community, where only the abundance was recorded.

Biomass was determined for the ground-dwelling and below-ground invertebrate communities. Following identification, all samples were dried to obtain a measurement of dry weight for the total invertebrate abundance per plot for each community. The excess alcohol was pipetted from the samples and the test tubes were placed in a drying cabinet for 72 hours at 60°C. Then specimens per plot, per sampling date, were weighed to a precision of 0.0001 g. The invertebrate voucher specimens are stored at CABI, Egham, UK.

### Data Analysis

Differences in the vegetation community composition, as well as temporal differences between invaded and uninvaded plots were analysed using principal response curves (PRC) [42], [43]. The PRC analysis is a special type of redundancy analysis (RDA) that allows for an evaluation of the temporal differences of the community. The PRC analysis assigns a weight to each taxon (taxon weight b_k_) which represents its contribution to the overall response of the PRC [44]. The uninvaded plots were treated as control plots and the invaded plots were assessed as deviations from the control. Sampling dates (months) were treated as repeated measurements and data for 2007 and 2008 were treated separately. Monte Carlo permutations [42] were used to test the overall significance of the PRC. To analyse differences in plant species richness between invaded and uninvaded plots, a two factor ANOVA was performed on the total number of plant species per plot, with invasion status and year as fixed effects. All percentage cover data were subjected to arc-sine transformation, averaged over the six sampled quadrats, per plot, to give an experimental plot mean, per year for each plant species.

Differences in the invertebrate community composition, as well as temporal differences between invaded and uninvaded plots were analysed using a PRC. The PRC analysis was applied to each invertebrate sampling method (community) and all replicates for each taxon per plot were pooled. All invertebrate abundance data were log transformed with the value of 1 added to each data point [42]. Differences in total invertebrate abundance, larval abundance, morphospecies richness, and invertebrate biomass between invaded and uninvaded plots were evaluated using a repeated measures ANOVA with invasion status and year as fixed effects.

To evaluate the effects of vegetation composition on invertebrate community composition multivariate statistics were used. The percentage cover of individual plant species were grouped into four categories: (1) *I. glandulifera* (2) trees and shrubs, (3) grasses, and (4) forbs. Although other non-native species were present, for example *I. capensis* Meerb., their cover was minimal in all plots and therefore did not warrant a separate non-native group. Only those invertebrate groups that showed a strong response in the PRC analysis were analysed [45]. A detrended correspondence analysis (DCA) was performed on the invertebrate data (response variable), for each sampling method, and the combined cover of the plant species in the four categories (explanatory variable). All gradient lengths were less than 4, invoking the adoption of a RDA. The significance of the results was tested using Monte Carlo permutation tests. Data for each sampling method (community) were analysed separately following log transformation of the data set.

Statistical analyses were performed with R version 2.12.2. [46]. All multivariate statistical analyses were conducted using the vegan package, version 1.17–10 [47].

We are very grateful to the owners of Harmondsworth Moor for allowing access to their land for the duration of this study.

## Results

### Vegetation

The vegetation community composition was significantly different between invaded and uninvaded plots (PRC: F_1,32_ = 8.66, *P*<0.05). Apart from the differences in *I. glandulifera* cover, the compositional changes were due mainly to differences in the percentage cover of seven native plant species (Table 1). In 2008, following a 63.4% reduction of *I. glandulifera* in the invaded plots, *Urtica dioica* L. and *Galium aparine* L. were the two native species to show the largest cover increases (Table 1). Plant species richness was similar between invaded and uninvaded plots (F_1,32_ = 0.43, *P*  = 0.51) and between years (F_1,32_ = 1.68, *P* = 0.21). Uninvaded plots had an average of 11.8±0.2 plant species per plot compared to 10.8±1.1 plant species in invaded plots.

**Table 1:** Summary of the difference in percentage cover of the seven native plant species which showed the strongest response in the principal response curve analysis.

Species	Form	Taxon weight (b_k_)	Difference between invaded and uninvaded plots		% change in invaded plots
			2007	2008	
*Agrostis stolonifera* L.	Grass	0.456	−92.1	−82.1	27.2
*Galium aparine* L.	Forb	−0.222	−49.1	153.8	413.7
*Holcus lanatus* L.	Grass	0.611	−86.5	−81.4	−36.2
*Juncus inflexus* L.	Grass	0.583	−98.2	−93.4	20.1
*Poa annua* L.	Grass	0.514	−95.1	−88.2	55.5
*Rubus fruticosus* agg. L.	Shrub	0.521	−67.5	−79.7	21.8
*Urtica dioica* L.	Forb	0.868	−89.1	−50.2	350.1

Shown are the species form, taxon weights (b_k_), difference in % cover in the invaded plots compared to the uninvaded plots for each year, and the difference in % cover in the invaded plots between years.

### Invertebrates

#### Foliage invertebrates

**Figure 1:**
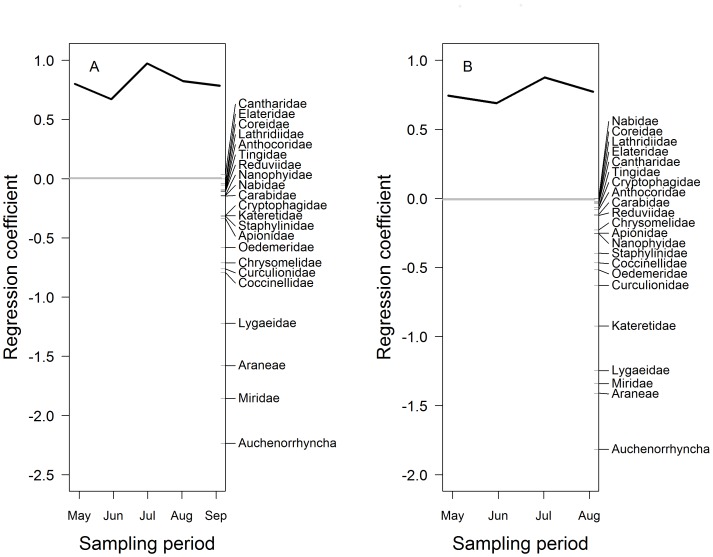
Principal response curves for the foliage invertebrate community. Figure (A) represents 2007 and figure (B) 2008. The uninvaded plots are expressed as the grey line (y = 0) and the black line is the response of the invertebrate community in the invaded plots, compared to the control (uninvaded), over time. The invertebrates groups on the third axis are ordered in their taxon weight corresponding to the y-axis. Both years are significant at *P*<0.05. For 2007, the first canonical axis explains 95.1% of the total variation where 10.32% is explained by time and 48.19% by treatment. For 2008, the first canonical axis explains 97.7% of the total variation where 7.06% was explained by time and 30.13% by treatment.

**Figure 2:**
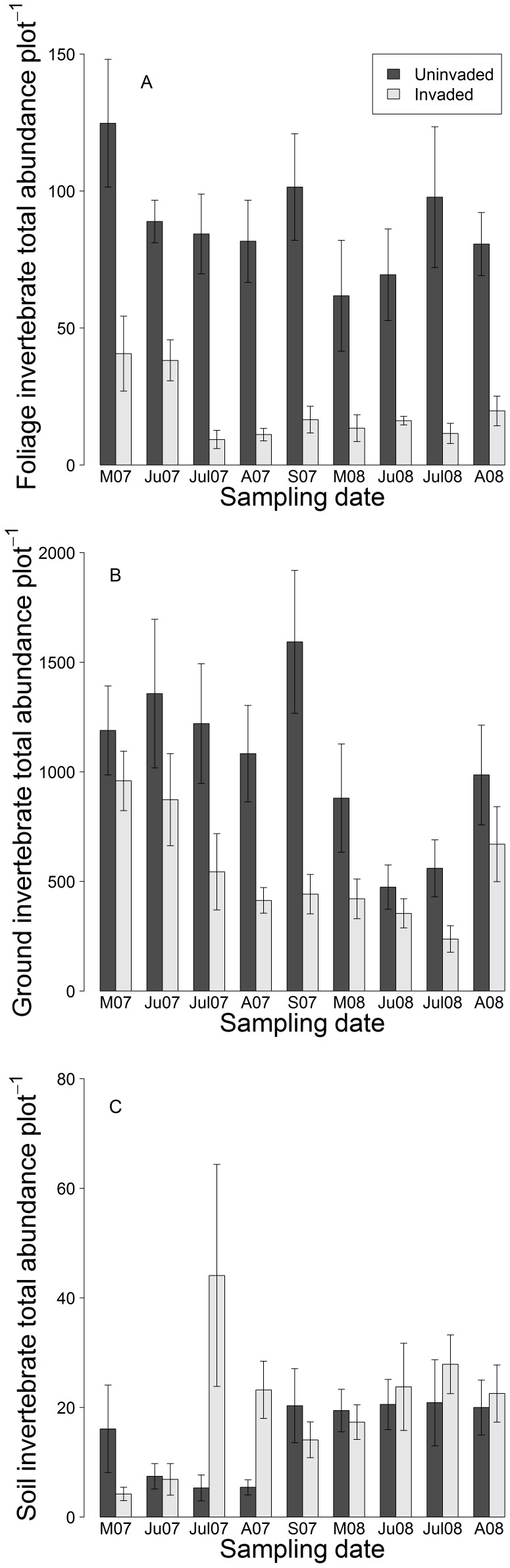
Total invertebrate abundance over time in invaded and uninvaded plots. Where figure (A) represents the foliage-dwelling community, (B) the ground-dwelling community and (C) the below-ground invertebrate community. Both the foliage-dwelling and ground-dwelling invertebrate communities had a significantly higher total abundance in uninvaded plots compared to invaded plots (*P*<0.001), whereas the below-ground invertebrate communities showed no difference.

The foliage invertebrate community structure showed significant differences between the invaded and uninvaded plots, as all invertebrate taxa were less abundant in the invaded plots compared to the uninvaded plots (2007: PRC: F_1,80_ = 47.88, *P*<0.05, Fig. 1a; 2008: PRC: F_1,64_ = 19.35, *P*<0.05, Fig. 1b, Table 2). Foliage invertebrate abundance was significantly lower in the invaded plots compared to the uninvaded plots for each sampling date (F_1,144_ = 110.06, *P*<0.001), and there was a significant temporal variation; total abundance was lower in 2008 compared to 2007 (F_1,144_ = 8.03, *P*<0.05) (Fig. 2a). All functional feeding groups were negatively associated with invaded plots for both years (2007: PRC: F_1,80_ = 93.62, *P*<0.05, 2008: PRC: F_1,64_ = 30.236, *P*<0.05, Table 3) but the largest differences between invaded and uninvaded plots occurred in July of both years.

**Table 2:** The taxon weights (b_k_) and difference in abundance of invertebrate groups from the foliage community.

		2007				2008			
		Taxon weight(b_k_)	Total abundance		Difference in abundance (%)	Taxon weight(b_k_)	Total abundance		Difference in abundance (%)
Group	Feeding Group		Invaded	Uninvaded			Invaded	Uninvaded	
**Arachnida**									
Araneae	Predator	−**1.578**	220	1039	−78.826	−**1.409**	189	651	−70.968
**Insecta**									
Coleoptera									
Apionidae	Herbivore	−0.33	2	32	–	−0.252	2	22	–
Cantharidae	Varied	0.035	7	5	–	−0.037	4	8	–
Carabidae	Varied	−0.145	12	23	–	−0.117	2	10	–
Chrysomelidae	Herbivore	−**0.711**	14	101	−86.139	−0.227	3	14	–
Coccinellidae	Predator	−**0.792**	15	94	−84.043	−0.467	0	33	–
Cryptophagidae	Varied	−0.313	1	21	–	−0.066	10	10	–
Curculionidae	Herbivore	−**0.762**	9	80	−88.75	−**0.629**	15	64	−76.563
Elateridae	Varied	−0.037	0	2	–	−0.028	0	1	–
Kateretidae	Herbivore	−0.313	3	27	–	−**0.923**	27	147	−81.633
Lathridiidae	Varied	−0.089	0	5	–	−0.023	0	1	–
Nanophyidae	Herbivore	−0.139	3	18	–	−0.253	0	17	–
Oedemeridae	Varied	−**0.581**	14	73	−80.822	−**0.516**	3	43	−93.023
Staphylinidae	Varied	−0.317	1	20	–	−0.395	1	21	–
**Hemiptera**									
Auchenorrhyncha	Herbivore	−**2.235**	648	2196	−70.492	−**1.815**	209	1322	−84.191
Heteroptera									
Anthocoridae	Predator	−0.098	1	11	–	−0.082	2	7	–
Coreidae	Herbivore	−0.053	0	3	–	0.001	0	0	–
Lygaeidae	Herbivore	−**1.221**	54	245	−77.959	−**1.246**	24	160	−85
Miridae	Herbivore	−**1.855**	34	354	−90.396	−**1.341**	48	209	−77.034
Nabidae	Predator	−0.143	1	11	–	0.001	0	0	–
Reduviidae	Predator	−0.111	4	10	–	−0.124	0	6	–
Tingidae	Varied	−0.109	0	7	–	−0.065	0	4	–

Taxon weights in bold indicate the groups that showed a strong response to the invaded plots. Differences in abundance are expressed as annual totals in the invaded plots to that of the uninvaded plots and are shown where the taxa showed a strong response to the invaded plots. The feeding group lists the dominant feeding group within the group.

**Table 3:** The taxon weights (b_k_) and difference in abundance of invertebrates categorised by functional feeding groups.

	Foliage-dwelling				Ground-dwelling				Below-ground			
Feeding group	2007		2008		2007		2008		2007		2008	
	Taxon weight(b_k_)	Difference in abundance (%)	Taxon weight(b_k_)	Difference in abundance (%)	Taxon weight(b_k_)	Difference in abundance (%)	Taxon weight(b_k_)	Difference in abundance (%)	Taxon weight(b_k_)	Difference in abundance (%)	Taxon weight(b_k_)	Difference in abundance (%)
Detritivore	–	–	–	–	−1.407	−75.23	−0.062	−34.149	−1.119	−2.542	−0.29	0.304
Herbivore	−2.665	−74.851	−2.272	−83.075	−1.897	−69.1	−1.595	−52.544	–	–	–	–
Predator	−1.885	−79.313	−1.636	−72.596	−1.223	−49.43	−1.047	−33.141	–	–	–	–
Varied	−1.234	−78.161	−1.05	−82.608	−1.146	−45.81	−1.551	−41.773	−1.962	184.52	−1.441	21.13

Differences in abundance are expressed as annual totals in the invaded plots compared to the uninvaded plots.

**Figure 3:**
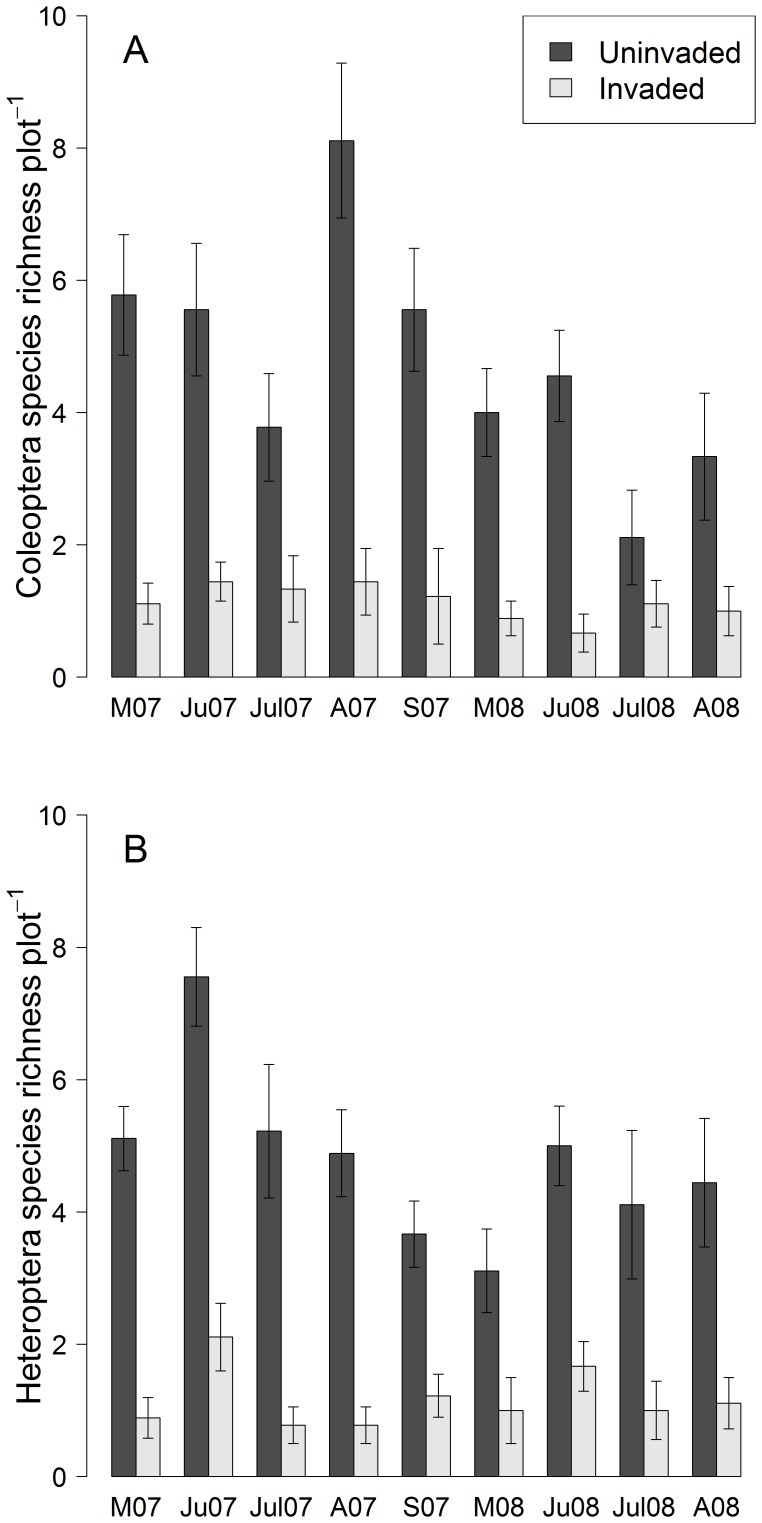
The difference between morphospecies richness between invaded and uninvaded plots. Figure (A) shows Coleoptera morphospecies richness and figure (B) shows Heteroptera morphospecies richness. Both Coleoptera and Heteroptera species richness was significantly higher in the uninvaded plots compared to the invaded plots (*P*<0.001).

In the foliage community, invaded plots had 64% less Coleoptera species than uninvaded plots (F_1,144_ = 102.21, *P*<0.001, Fig. 3a). Similarly, Heteroptera species richness was 58% lower in invaded plots (F_1,144_ = 146.83, *P*<0.001, Fig. 3b). Coleoptera species richness was positively correlated with native plant species richness for all plots (r = 2.37, n = 18, *P*<0.05), however, there was no correlation in just the invaded plots (r = 0.99, n = 9, *P* = 0.35). There was no correlation between Heteroptera richness and native plant richness when evaluating both invaded and uninvaded plots (r = 0.92, n = 18, *P* = 0.51) or just the invaded plots (r = 0.97, n = 9, *P* = 0.67).

#### Ground-dwelling invertebrates

**Figure 4:**
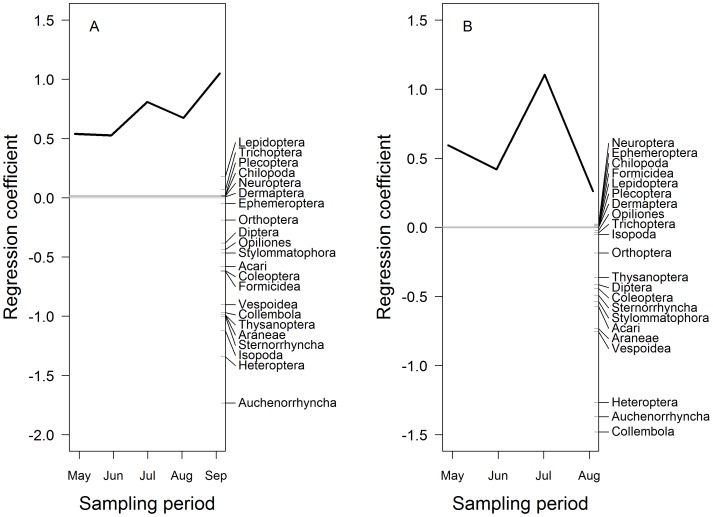
Principal response curves for the ground-dwelling invertebrate community. Figure (A) represents 2007 and figure (B) 2008. The uninvaded plots are expressed as the grey line (y = 0) and the black line is the response of the invertebrate community in the invaded plots, compared to the control (uninvaded), over time. The invertebrates groups to the right of the graphic are ordered in their taxon weight corresponding to the y-axis. Both years are significant at *P*<0.05. For 2007, the first canonical axis explains 82.2% of the total variation where 18.51% was explained by time and 13% by treatment. For 2008, the first canonical axis explains 78.1% of the total variation where 41% was explained by time, and 6.4% by treatment.

For the ground-dwelling invertebrate community composition, Formicidae, Coleoptera, Acari, Isopoda, Thysanoptera, Sternorrhyncha, Vespoidea, Araneae, Collembola, Heteroptera, and Auchenorrhyncha all showed a lower abundance in the invaded plots in 2007 compared to the uninvaded plots (PRC: F _1,80_ = 11.27, *P*<0.05, Fig. 4a, Table 4). However, in 2008, only Acari, Araneae, Auchenorrhyncha, Collembola, Heteroptera Vespoidea and Stylommatophora were rarer in the invaded plots (PRC: F_1,64_ = 9.88, *P*<0.05, Fig. 4b, Table 4). In both years, the community response was stronger in July than in the preceding months and in August, but the difference between invaded and uninvaded plots was also comparatively strong in September 2007. Herbivorous taxa showed the strongest negative response in the invaded plots for both 2007 (PRC: F_1,80_ = 23.64, *P*<0.05) and 2008 (PRC: F_1,64_ = 10.79, *P*<0.05) (Table 3). None of the invertebrate groups showed a strong positive association with *I. glandulifera* in the invaded plots. Total invertebrate abundance was consistently higher in the uninvaded plots (F_1,144_ = 27.81, *P*<0.001) and there was significant temporal variation: total abundance was lower in 2008 compared to 2007 (F_1,144_ = 20.78, *P*<0.001, Fig. 2b). Total invertebrate biomass was similar between invaded and uninvaded plots (F_1,144_ = 3.19, *P* = 0.08).

**Table 4:** The taxon weights (b_k_) and difference in abundance of invertebrate groups in the ground- and below-ground communities.

		Ground-dwelling				Below-ground		
		2007		2008		2007		2008
Group	Feeding Group	Taxon weight(b_k_)	Difference in abundance (%)	Taxon weight(b_k_)	Difference in abundance (%)	Taxon weight(b_k_)	Difference in abundance (%)	Taxon weight(b_k_)
**Arachnida**								
Acari	Varied	−**0.579**	−66.722	−**0.574**	−5.567	−**1.026**	106.667	−0.223
Araneae	Predator	−**0.989**	−50.061	−**0.733**	−34.645	–	–	–
Opiliones	Varied	−0.436	–	−0.021	–	–	–	–
**Chilopoda**	Predator	0.018	–	0.017	–	–	–	–
**Clitellata**								
Haplotaxida	Detritivore	–	–	–	–	−**0.724**	162.162	−0.252
**Diplopoda**	Detritivore	–	–	–	–	−0.387	–	0.121
**Gastropoda**								
Stylommatophora	Herbivore	−0.461	–	−**0.537**	−59.000	–	–	–
**Entognatha**								
Collembola	Varied	−**0.969**	−44.289	−**1.479**	−52.305	−**1.624**	569.23	−1.566
**Insecta**								
Coleoptera	Varied	−**0.614**	−36.727	−0.443	–	−**0.915**	151.02	0.29
Dermaptera	Varied	0.008	–	0.001	–	–	–	–
Diptera	Varied	−0.381	–	−0.414	–	–	–	–
Ephemeroptera	Adults non-feeding	−0.048	–	−0.025	–	–	–	–
**Hemiptera**								
Auchenorrhyncha	Herbivore	−**1.732**	−75.614	−**1.37**	−58.443	–	–	–
Heteroptera	Varied	−**1.343**	−71.516	−**1.267**	−59.011	–	–	–
Sternorrhyncha	Herbivore	−**1.002**	−51.412	−0.492	–	–	–	–
**Hymenoptera**								
Formicidae	Varied	−**0.622**	−83.659	−0.009	–	−0.142		0.117
Vespoidea	Predator	−**0.902**	−48.560	−**0.755**	−30.389	–	–	–
Lepidoptera	Herbivore	0.182	–	−0.009	–	–	–	–
Neuroptera	Predator	0.015	–	0.025	–	–	–	–
Orthoptera	Herbivore	−0.188	–	−0.185	–	–	–	–
Plecoptera	Herbivore	0.022	–	0.001	–	–	–	–
Thysanoptera	Varied	−**0.987**	−81.206	−0.362	–	0.095	–	−0.05
Trichoptera	Varied	0.067	–	−0.038	–	–	–	–
**Malacostraca**								
Isopoda	Detritivore	−**1.122**	−75.23	−0.051	–	−0.131	–	0.307

Taxon weights in bold indicate the groups that showed a strong response to the invaded plots. Differences in abundance are expressed as annual totals in the invaded plots to that of the uninvaded plots and are shown where the taxa showed a strong response to the invaded plots. The feeding group lists the dominant feeding group within the group. Differences in abundance are not shown for the 2008 below-ground taxa as none of the groups showing a strong response to the invaded sites.

#### Below-ground invertebrates

**Figure 5:**
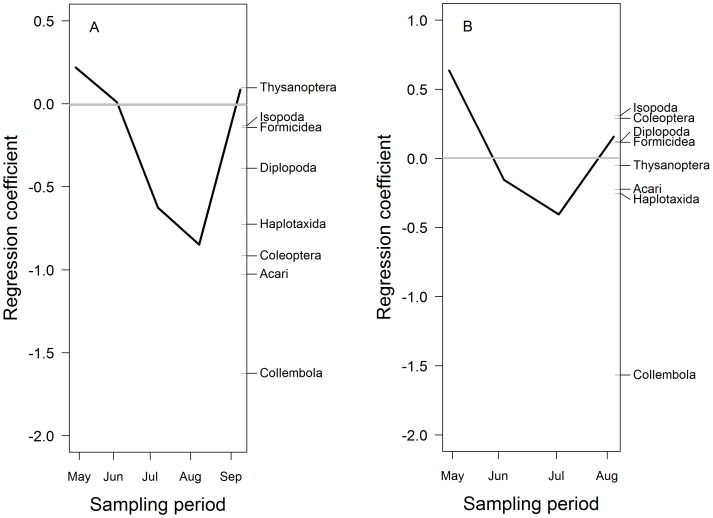
Principal response curves for the below-ground invertebrate community. Figure (A) represents 2007 and figure (B) 2008. The uninvaded plots are expressed as the grey line (y = 0) and the black line is the response of the invertebrate community in the invaded plots, compared to the control (uninvaded), over time. The invertebrates groups on the third axis are ordered in their taxon weight corresponding to the y-axis. The 2007 data set shows a significant shift in the invertebrate community between the invaded and invaded plots (*P*<0.05) where the first canonical axis explains 59.91% of the total variation where 12.82% was explained by time and 7.56% by treatment. There was no significant shift in 2008 but the graphic is shown for completeness.

In contrast to the foliage- and ground-dwelling communities, the difference seen in the below-ground invertebrate community in 2007 (PRC: F_1,80_ = 8.74, *P*<0.05, Fig. 5a) was largely a result of a higher abundance of Collembola and detritivores in the invaded stands in the summer months of June and July (PRC: F_1,80_ = 13.8, *P*<0.05). Collembola showed the highest response to *I. glandulifera* and the population was 5 times of the abundance in the uninvaded plots (Table 4). In 2008, the below-ground invertebrate community composition was similar in invaded and uninvaded plots (PRC: F_1,64_ = 1.54, *P* = 0.8, Fig. 5b). In the below-ground community, invertebrate abundance (F_1,144_ = 1.73, *P* = 0.18, Fig. 2c), and total invertebrate biomass (F_1,144_ = 3.68, *P* = 0.56) were similar in invaded and uninvaded plots. However, the abundance of invertebrate larvae was significantly higher in the invaded plots (F_1,144_ = 7.904, *P*<0.05) (invaded: 3.012±0.35, uninvaded: 2.037±0.371).

### Relationship between Vegetation and Invertebrate Community Composition

**Figure 6:**
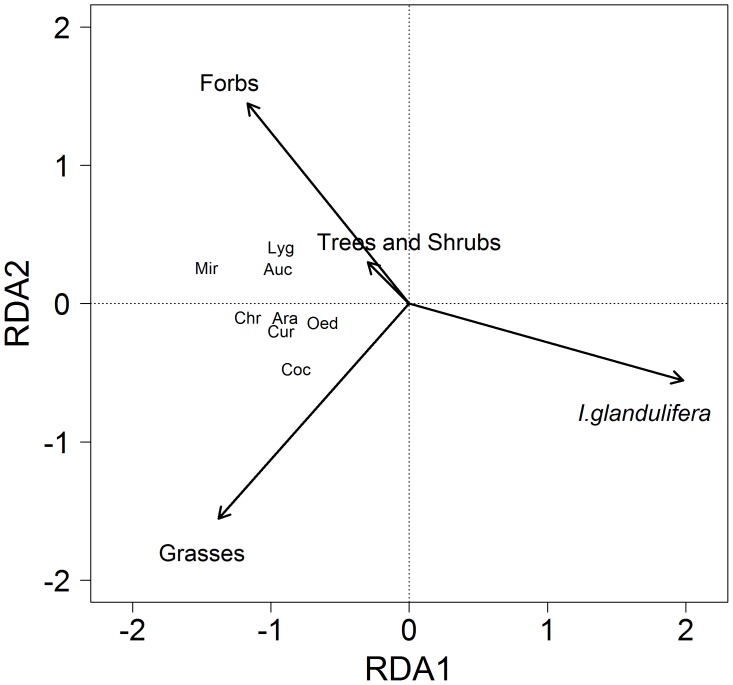
Biplot of the percentage cover of vegetation and invertebrate abundance for the 2007 foliage-dwelling community. The total variation explained is 69.55%. Axis 1 explains 63.64% and axis 2 explains 4.27% (*P*<0.05). Plot labels are: Ara: Araneae, Auc: Auchenorrhyncha, Chr: Chrysomelidae, Coc: Coccinellidae, Cur: Curculionidae, Oed: Oedemeridae, Lyg: Lygaeidae, Mir: Miridae.

**Figure 7:**
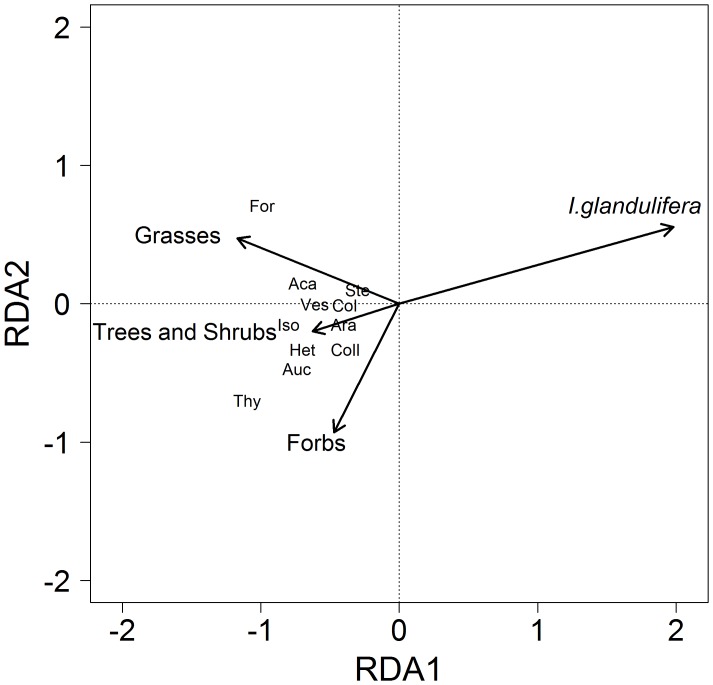
Biplot of the percentage cover of vegetation and invertebrate abundance for the 2007 ground-dwelling community. The total variation explained is 48.8%. Axis 1 explains 40.4% and axis 2 explains 7.1% (*P*<0.05). Plot labels are: Aca: Acari, Ara: Araneae, Auc: Auchenorrhyncha, Col: Coleoptera, Coll: Collembola, For: Formicidae, Het: Heteroptera, Iso: Isopoda, Ste: Sternorrhyncha, Thy: Thysanoptera, Ves: Vespoidea.

There was a significant relationship between the foliage invertebrate community and the vegetation composition in 2007 (F_4,13_ = 7.42, *P*<0.05), but not in 2008 (F_4,13_ = 1.36, *P* = 0.24). In 2007, the majority of variation explained in the composition of the invertebrate groups was related to the variation of *I. glandulifera* cover (Fig. 6). Similarly, there was a significant relationship between the ground-dwelling invertebrate community composition and cover of the four plant groups in 2007 (F_4,13_ = 3.11, *P*<0.05), and an indication of a significant relationship in 2008 (F_4,13_ = 1.95, *P* = 0.052). In 2007, the majority of variation in the ground-dwelling invertebrate community composition was related to the percentage cover of *I. glandulifera* (Fig. 7). There was no relationship between the below-ground invertebrate community composition and vegetation composition in 2007 (F_4,13_ = 1.29, *P* = 0.26) or 2008 (F_4,13_ = 0.11, *P* = 0.8).

## Discussion

This study shows that the above- and below-ground invertebrate communities respond differently to the presence of *I. glandulifera*. Whereas in the foliage- and ground-dwelling communities the invertebrate groups in invaded plots showed reduced abundance compared to the uninvaded plots, the below-ground community appeared to be more resilient to the occurrence of *I. glandulifera*. The reductions were more pronounced for the foliage-dwelling invertebrates compared to the ground-dwelling invertebrates and this is potentially due to the availability of food in the canopy of the invaded plots compared to resources at ground level. Similar contrasts between the foliage- and ground-dwelling invertebrate communities have been shown when studying the invertebrates associated with perennial species, for example, the non-native grass species *Arundo donax* L. in California, USA [48].

Herbivorous invertebrates showed a consistent negative response to *I. glandulifera* in the foliage- and ground-dwelling communities, and this may be due to *I. glandulifera* being unpalatable to the majority of invertebrate species in its introduced range. Chrysomelidae and Curculionidae (Coleoptera), and Lygaeidae and Miridae (Heteroptera) were all negatively affected by *I. glandulifera* in the foliage community in 2007 and the two latter families in 2008. Auchenorrhyncha abundance was consistently negatively affected by *I. glandulifera* in both the foliage- and ground-dwelling communities during both years. Herbivore, or more generally invertebrate abundance and diversity, is influenced by vegetation biomass and community composition [45], [49]. The dominance of *I. glandulifera* in the invaded plots alters the natural vegetation composition and potentially reduces the biomass of native plant species by competing with these species for light and soil nutrients. Heteroptera and Coleoptera both showed lower species richness in the foliage community in the invaded plots, and the positive correlation of Coleoptera species richness to native plant species richness highlights the dependence of Coleoptera on the native plant community.

Some vigorous invaders, for example non-native plant species of the genus *Fallopia*, almost permanently simplify an invaded habitat, in terms of structural diversity leading to beneficial niches for exploitation by predatory invertebrate groups [50]. However, our data suggest that this is not true for all invasive species. In our study, spiders (Araneae) were consistently negatively associated with the invaded plots for both years. Spiders may potentially be more sensitive to the annual fluctuations in cover of *I. glandulifera* than the more mobile (flying) prey species, thus preferring undisturbed habitats [51]. All predatory Heteroptera were unaffected by the presence of *I. glandulifera* though the low abundance of these groups in both the invaded and uninvaded plots does not allow for a quantitative comparison.

Coccinellidae (Coleoptera) showed a strong negative response to the invaded plots in 2007 though in 2008 the response was not as obvious. The lower abundance of Coccinellidae in the invaded plots may be a direct cause of a lower abundance of small prey groups like Sternorrhyncha in the above-ground communities. The sampling methods utilised in the above-ground communities (aerial suction sampler and Vortis suction sampler) may have a decreased sampling efficiency in dense vegetation or vegetation with increased aerial structure (invaded plots) [40]. However, when present in the uninvaded plots, *Phalaris arundinacea* L. and thickets of *Rubus fruticosus* agg. L. could reach heights comparable with *I. glandulifera* in invaded plots, suggesting that vegetation structure was not a reason for the observed differences between invaded and uninvaded plots.

Our results show that although the cover of *I. glandulifera* was lower in the invaded plots in 2008, the invertebrate communities followed a similar seasonal fluctuation for both years. In both the foliage- and ground-dwelling invertebrate communities, the largest difference between invaded and uninvaded plots generally occurred in July. The timing of these differences coincided with peak vegetation biomass, and the most responsive groups were herbivores. The abundance of herbivores was lower in invaded plots; however, the seasonal response seems to be the result of lower (relative) abundance of host plants in invaded plots. In the ground-dwelling community, in 2007, there was also a large response to the invaded plots in September, which appears also to be due to herbivores. This response could be explained by the dieback of the limited cover of native plants, and their reduced food quality, in the invaded plots due to the onset of the autumn.

In the ground-dwelling community, ten invertebrate groups showed no response to the presence of *I. glandulifera* and 60% of these were flying groups like Diptera and Lepidoptera. At least some members of these groups would use the structure of *I. glandulifera* as resting sites without feeding on the plant. When studying the impact of an introduced palm species (*Phoenix canariensis* Chabaud) along the San Diego River in California, USA, Talley et al. [22] showed that there was no impact of the palm on the ground-dwelling invertebrate community when compared to native stands of willow (*Salix lasiolepis* Benth.). What our study and that of Talley et al. [22] highlights is that in contrast to the foliage invertebrate community, ground-dwelling invertebrates are not solely reliant on live plants for food and development and so might not be affected by changes in vegetation composition caused by an invasive plant.

The abundance of detritivores was significantly lower in the invaded plots at ground level in 2007. Decomposition by detritivores is a fundamental ecosystem process linking the above- and below-ground communities, and *I. glandulifera* may disrupt this by incorporating an increased amount of organic matter into the ecosystem compared with natural vegetation. Temporal impacts on ecosystem functioning may be caused by the slower breakdown of organic material from non-native species compared to native species. *Impatiens glandulifera* has a similar chemical concentration of nitrogen, phosphorus, and potassium to *F. japonica* [35], which has been shown to have slower decomposition rates compared to native species. Indeed, the previous season’s stems of *I. glandulifera* are frequently visible as dried material in the spring and early summer months of the following season. Changes in the rate and amount of decomposition of organic matter in invaded plots may lead to food material becoming available at different times of the year compared to uninvaded plots.

In contrast to the above-ground communities, in the below-ground community, detritivores and taxa comprising of species that feed on various prey were more abundant in the invaded plots in 2007. Possible explanations for this could be the more generalist feeding habits of below-ground invertebrates [52], [53] and lower levels of host specificity of organisms in the soil [54]. In 2008, following the reduced cover of *I. glandulifera* in the invaded plots, the below-ground community alone showed no change in invertebrate composition between the invaded and uninvaded plots. Although these results suggest that the presence of *I. glandulifera* may affect the below-ground community differently (positively) to that of the above-ground communities, it is hard to determine signs of recovery in a two year study. It is quite possible that our results simply reflect inter-annual variation in the invertebrate community.

Collembola responded positively to invaded plots in 2007 where the total abundance was 569% of that in the uninvaded plots. The increased root mass in an area invaded by *I. glandulifera* may act to increase the amount of food available to Collembola and detritivores throughout the growing season, and beyond as the roots break down. Brown and Gange [54] suggest that root herbivores are affected more by root quantity than quality, which may suggest why Collembola are more frequent in the invaded plots. An alternative explanation is that the dense stands of *I. glandulifera* act to alter the soil moisture content compared to natural vegetation providing a more favourable habitat for Collembola in the dry summer months. This is expressed by the similar pattern in the response of the below-ground invertebrate community in both years, where the abundance increased in the drier summer months of July and August, which was driven largely by increased numbers of Collembola.

The population dynamics of *I. glandulifera*, as an annual species, results in fluctuating cover year on year throughout its introduced range, and may explain why the species is thought to have variable impacts on the habitats it invades [6], [55]. In our study, although *I. glandulifera* significantly decreased in cover in the second year of the study, the above-ground invertebrate communities did not significantly respond. In the foliage-dwelling community, none of the invertebrate groups increased in abundance, and in the ground-dwelling community, only 45% of the invertebrate groups increased in 2008. The lack of an overall response may be due to the quality and quantity of (native) vegetation in the invaded plots compared to the uninvaded plots [56]. Although plant species richness remained relatively constant in both invaded and uninvaded plots between years, the shading of native species by *I. glandulifera* may act to reduce native plant fitness and subsequent seed set, which in turn may lead to reduced niche availability for the invertebrate community even with a reduction in *I. glandulifera.* Residual indirect effects as a result of the historic occurrence of *I. glandulifera* may influence the performance and quality of native plant species in the invaded plots [12], [57]. Our data does suggest that any recovery of invertebrate communities after weed removal will be a slow process.

The reduced abundance and displacement of invertebrate groups in invaded stands may have potential consequences at higher trophic levels. *Impatiens glandulifera* is present in riparian systems throughout the UK where the most recent estimates suggest it occupies some 13% of English and Welsh rivers [58]. If *I. glandulifera* is having impacts on invertebrate communities throughout the invaded region, similar to those we have shown in this study, this may have significant impacts on invertebrate populations in riparian systems on a nationwide scale, which would potentially feed through the system, affecting higher trophic levels. Research into the impact of *Solidago* species on grassland birds in Eastern Europe showed that invasion significantly reduced species richness by reducing food availability [59]. When studying the impact of *Salix* x *repens* L., an invasive riparian tree species in eastern Australia, Holland-Clift et al. [60] showed that sites dominated by this species had reduced bird diversity. Meanwhile in the same region, Greenwood et al. [61] showed *Salix* x *repens* reduced invertebrate community composition and abundance.

Non-native invasive plant species have been shown to have an impact on ecosystem functioning and processes by displacing plant species and functional invertebrate groups in the community [62]. Understanding the impacts of *I. glandulifera* on different parts of the ecosystem is essential to understand the impacts in the context of these processes. Within the invaded plots, differences in the response of the invertebrate communities varied, where a general decrease in the negative response was observed moving from the canopy to below ground. The potential influx of invertebrates into the below-ground community, as a result of *I. glandulifera* invasion, and the displacement of functional groups in the above-ground communities, can potentially lead to a habitat less biologically diverse than surrounding native communities. This study showed that the above-ground invertebrate communities associated with *I. glandulifera* are impoverished, compared to those of adjacent natural vegetation. The lower herbivore abundance can potentially influence the abundance of below-ground herbivores, as the amount of subterranean biomass of the invasive remains largely unregulated in invaded plots [63]. The indirect impacts of the invasion of *I. glandulifera* on the performance of native plant species within, and around, invaded stands needs further exploration, but potentially the presence of *I. glandulifera* may act to reduce the fitness of native plant species by reducing the resource allocation from below ground to above ground.

In conclusion, whereas most impact studies on *I. glandulifera* have focused on the impacts on native vegetation, here we show that *I. glandulifera* has a negative impact on associated invertebrate communities, and this is more pronounced in above-ground communities. Although *I. glandulifera* is managed throughout the UK, there is still little focus on the restoration of the degraded habitats with native plant species and further research is needed to evaluate how restoring a habitat following the removal of *I. glandulifera* could promote native biodiversity at higher trophic levels.
